# An efficient, high‐throughput method for the simultaneous exposure of drought stress and bacterial infection in plants

**DOI:** 10.1002/aps3.11399

**Published:** 2020-12-01

**Authors:** Aanchal Choudhary, Muthappa Senthil‐Kumar

**Affiliations:** ^1^ National Institute of Plant Genome Research Aruna Asaf Ali Marg New Delhi 110067 India

**Keywords:** *Arabidopsis thaliana*, bacteria, combined stress, drought, *Pseudomonas syringae*

## Abstract

**Premise:**

We developed a systematic protocol for the easy, high‐throughput, qualitative, and quantitative assessment of the patho‐morphological, physiological, and molecular responses of *Arabidopsis thaliana* plants simultaneously subjected to drought and bacterial infection. This approach will assist studies elucidating plant adaptation strategies to combat combined stresses.

**Methods and Results:**

Plants were grown in small screw‐capped containers, individual pots, or pot strips. Watering was withheld from 30‐day‐old plants, which were subsequently infected with *Pseudomonas syringae* pv. *tomato* DC3000 using a dip inoculation. The natural development of both drought and bacterial infection was successfully and rapidly replicated in large numbers of plants, which is difficult to achieve with existing protocols.

**Conclusions:**

Our protocol offers a simple, low‐cost, high‐throughput strategy for the rapid and easy bacterial infection of large numbers of plants. It can be used in large‐scale mutant and ecotype screenings under combined stresses and for other foliar pathogens in different plant species.

Drought is an important stress factor affecting plant growth and development and influencing their responses to bacterial pathogens (Ramegowda and Senthil‐Kumar, 2015), both positively (Ramegowda et al., 2013; Gupta et al., 2016; Sinha et al., 2016) and negatively (Mohr and Cahill, 2003; Choi et al., 2013; Dossa et al., 2017). In the field, plants frequently encounter water deficits and bacterial pathogens simultaneously, and a plant’s response to either stress is greatly affected by the severity, duration, and timing of the other stress factor (Ramegowda et al., 2013; Gupta et al., 2016). The co‐occurrence of drought and bacterial stresses is expected to become more prevalent because of global climate change and growing water scarcity. Because plant responses to combined stresses are specific to the circumstances involved, single‐stress studies are insufficient for drawing accurate conclusions, necessitating combined‐stress studies, which are currently limited.

A major bottleneck in undertaking combined‐stress studies is the unavailability of an efficient platform for plant phenotyping. This is because the development of a water deficit (drought) and its perception by plants are gradual, whereas bacterial infection and disease development occur quickly. Simultaneously inflicting stresses at a similar intensity thus becomes difficult. Although several studies have successfully co‐imposed drought and bacterial stress, the utility of the methods used is limited by several factors (Ramegowda et al., 2013; Gupta et al., 2016; Sinha et al., 2016; Dixit et al., 2019). The well‐established *Arabidopsis thaliana–Pseudomonas syringae* pathosystem has been successfully used in combined‐stress studies, and infection protocols are available for *P. syringae* (Katagiri et al., 2002; Yao et al., 2013; Rufián et al., 2019). The most routinely used syringe infiltration method involves injecting bacteria directly into the apoplast, bypassing the natural infection pathway through the stomata (Tornero and Dangl, 2001; Katagiri et al., 2002; Liu et al., 2015). Besides being time consuming and labor intensive, syringe infiltration requires expertise to minimize the mechanical damage to soft leaf tissue during its use. More importantly, the method cannot be used for studying pre‐invasive defenses such as stomatal and cuticular defenses (Melotto et al., 2008; Panchal et al., 2016), two important plant defense mechanisms against both bacteria and drought (Melotto et al., 2017). The flood‐inoculation method for *A. thaliana* (L.) Heynh. seedlings grown on Murashige and Skoog plates cannot be used for soil‐grown plants (Ishiga et al., 2011; Dixit et al., 2019) and is thus limited in its use. Furthermore, it is challenging to avoid plant damage when spraying the abaxial surface of leaves in the spray‐inoculation method (Katagiri et al., 2002), and the use of a vacuum chamber is also cumbersome for high‐throughput assays.

Several approaches have also been used for the imposition of drought in combined‐stress studies, such as withholding irrigation (Ramegowda et al., 2013; Gupta et al., 2016) and using polyethylene glycol (PEG) (Dixit et al., 2019); however, drought stress induced by PEG occurs rapidly, failing to simulate the true scenario of a gradually developing water deficit. Plants adopt several strategies to sustain their growth under drought, minimizing water loss even under reduced soil moisture; thus, these nuances and the effect of a simultaneously occurring stressor can go unnoticed if the drought stress is imposed rapidly. Moreover, osmolytes such as PEG decrease the water potential of the plant growth medium and thereby disrupt water absorption by the roots without affecting subsequent transpiration loss and sensing/signaling, which is the primary trigger for drought responses in natural conditions. The existing methods are therefore limited in their ability to simulate the actual stress scenario and instead rapidly impose stresses on the plants.

In order to address these limitations, we devised a systematic methodology for the effective co‐imposition of drought and bacterial stress using methods that closely approximate the natural mode of stress occurrence with a minimal infliction of any mechanical injury to the plant. *Arabidopsis thaliana* plants were subjected to water withholding, and drought was allowed to progress gradually throughout the experiment; subsequently, a bacterial infection was performed by dip inoculation. This methodology can be used for studying both pre‐ and post‐invasive defense strategies against foliar pathogens in several plant species and could benefit a wide research community. The approach is quick and inexpensive and is amenable to scaling up, which is difficult to achieve with currently existing protocols.

## METHODS AND RESULTS

### Plant growth conditions

**Figure 1:**
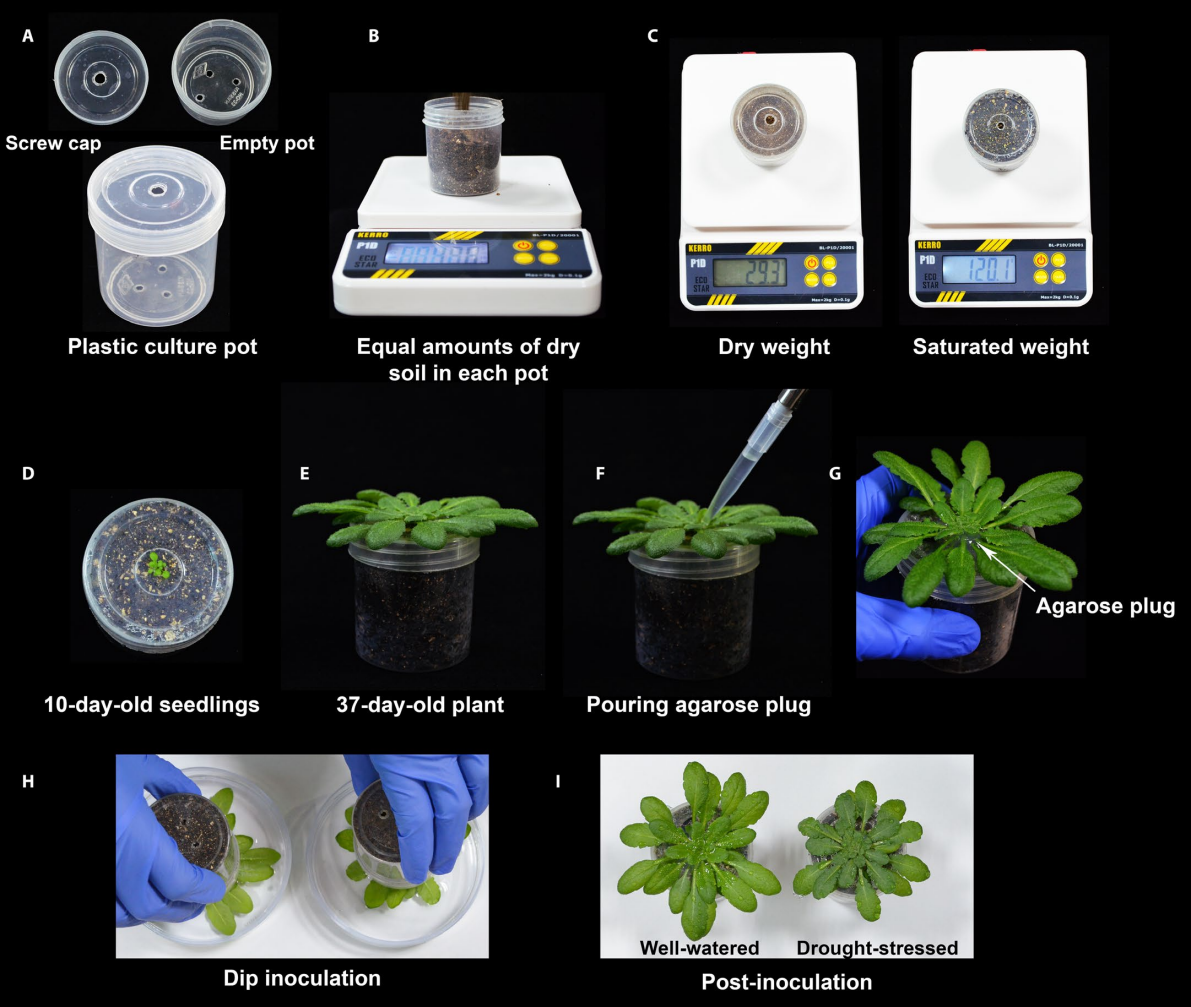
Preparation of pots with screw caps for bacterial inoculation under combined stress. (A) An empty plastic container used for growing plants, with three holes at the bottom to allow water absorption and one hole at the top for sowing seeds. (B) The container is filled with air‐dried potting mix. (C) Weight of the container with dry potting mix (left) and water‐saturated potting mix (right). (D) Ten‐day‐old seedlings emerging from the top. All but one seedling is removed at this stage. (E) A 37‐day‐old plant used for the experiment. (F, G) The potting mix around the plant is plugged with 2% agarose immediately before inoculation. (H) The rosette of the plants is dipped into the bacterial suspension. (I) Well‐watered (left) and drought‐stressed (right) plants immediately after bacterial infection.

**Figure 2:**
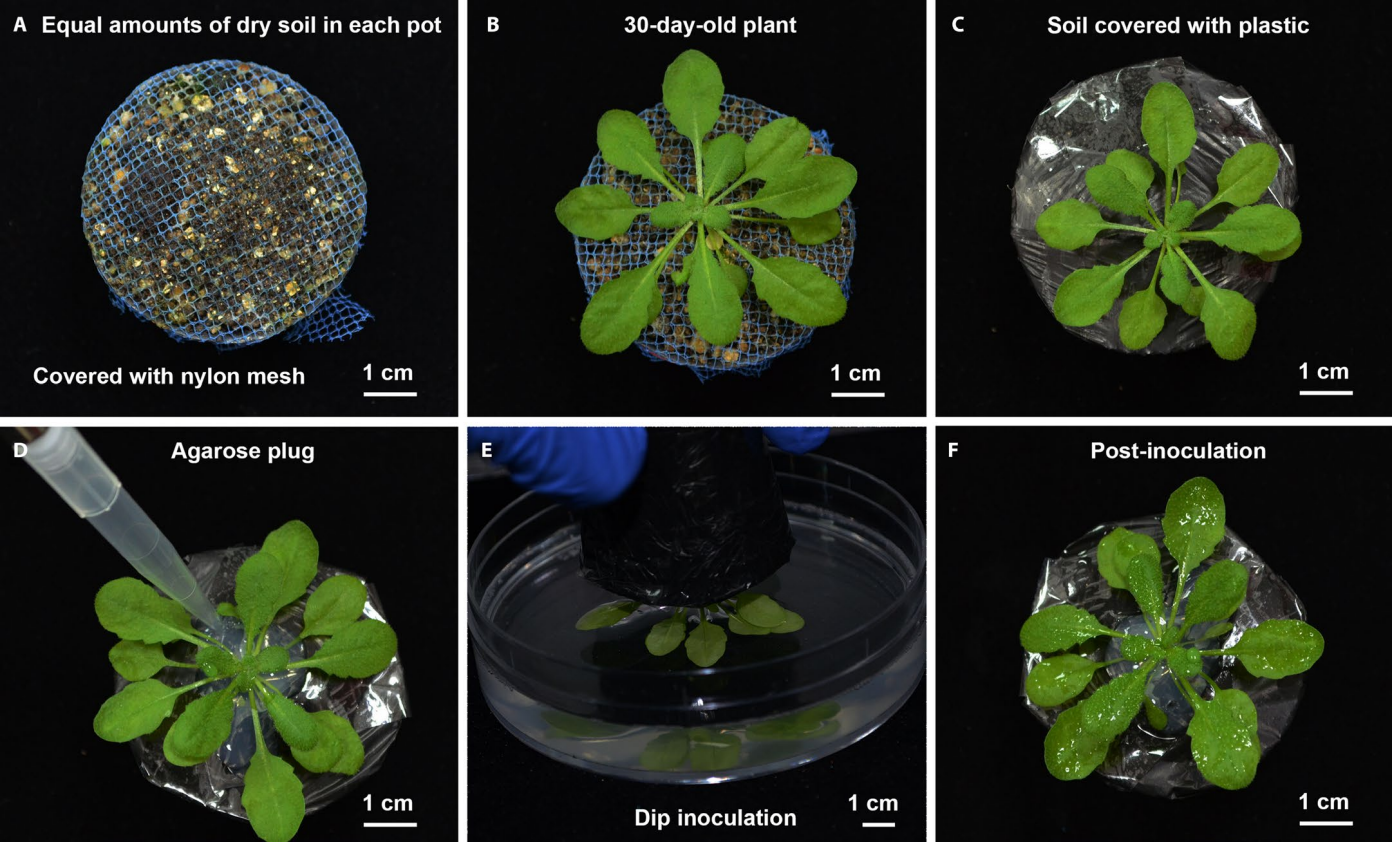
Preparation of single open pots for bacterial inoculation under combined stress. (A) An empty plastic pot is filled with air‐dried potting mix, covered with a nylon mesh, and saturated with water. (B) A 30‐day‐old plant emerging from the nylon screen. (C) The potting mix around the plant is covered using thin strips of plastic bags. (D) The potting mix around the plant is plugged with agarose to seal any space and prevent the entry of the bacterial suspension during inoculation. (E) The rosette of the plant is dipped into the bacterial suspension. (F) The plant’s surface is completely covered with the bacterial suspension.

**Figure 3:**
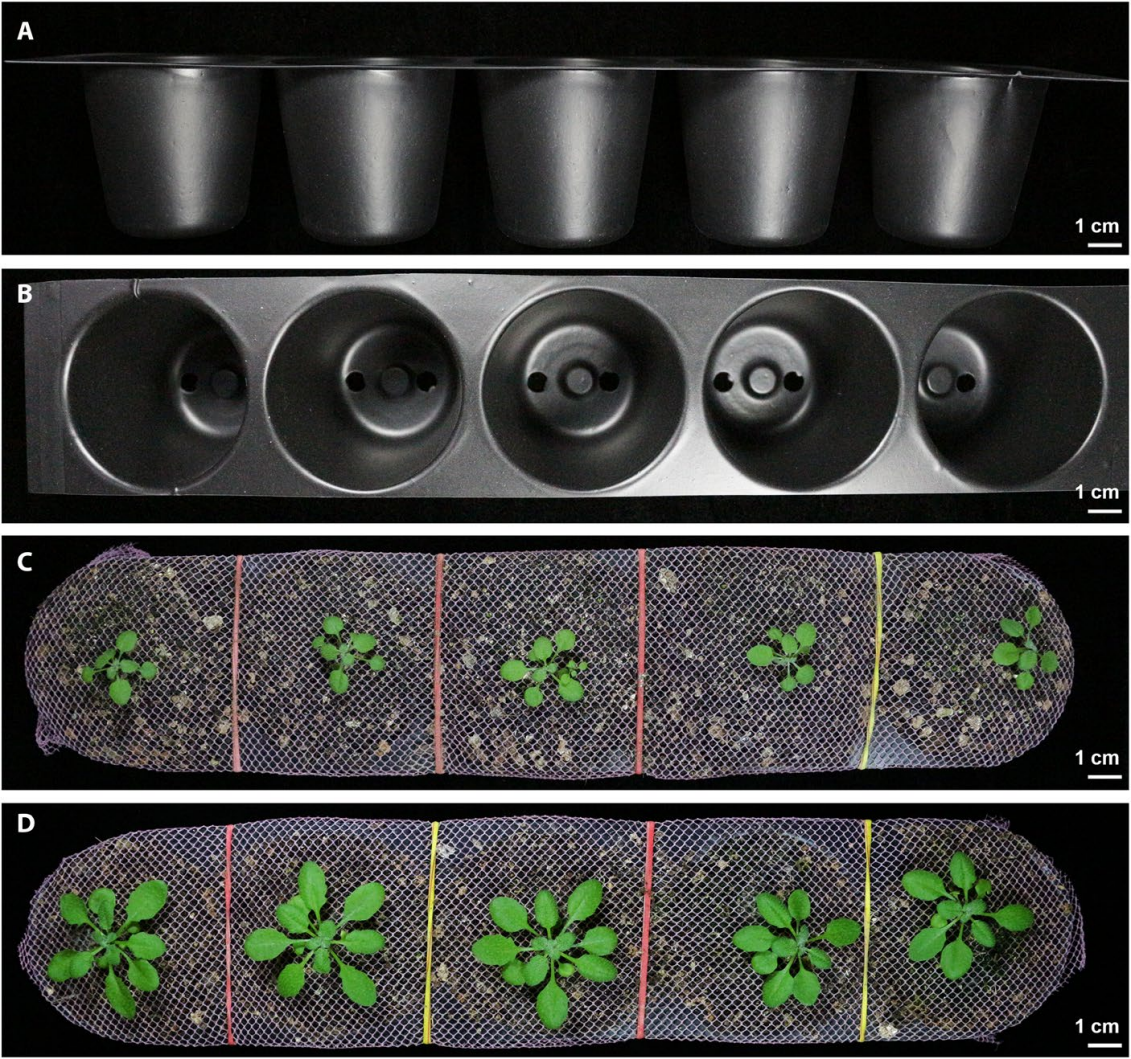
Preparation of strip trays for bacterial inoculation under combined stress. (A) An empty five‐pot strip tray, side view. (B) Top view of the strip tray. Each pot will be filled with an equal amount of dried potting mix, covered with a nylon mesh, and saturated with water. (C, D) Plants emerging from the nylon screen at 15 days (C) and 25 days (D).

*Arabidopsis thaliana* ecotype Columbia (Col‐0, CS70000), procured from the Arabidopsis Biological Resource Center (The Ohio State University, Columbus, Ohio, USA), was used in this study. Three types of platforms were used for growing plants (Appendix 1): (1) plants were grown in small individual screw‐capped plastic containers (6.0 cm height, 5.2 cm diameter) (Fig. 1), with three holes punched in the bottom of the containers for water absorption and one hole punched in the cap to sow seeds; (2) plants were grown in individual open plastic pots (4.5 cm height, 5 cm diameter) (Fig. 2); and (3) plants were grown in five‐pot strip trays (4.5 cm height, 25 cm length) (Fig. 3).

The containers, pots, and strip trays were filled with equal amounts of sterile potting mix (air‐dried agropeat and vermiculite, 3 : 1 v/v; Varsha Enterprises, Jayanagar, Bengaluru, Karnataka, India), after which they were capped shut and the open pots and strip trays were covered with a nylon mesh to prevent the potting mix and plant from falling out during the dip inoculation. After noting the initial weight of each type of container filled with completely dry potting mix (dry weight [DW]), they were placed in flat trays filled with 0.5× Hoagland solution (HiMedia, Mumbai, India) for 2 h. When the potting mix was thoroughly soaked, the excess water was drained off and each container/pot/strip tray was reweighed (saturated weight [SW]). The seeds were directly sown into the pots, covered, and stratified at 4°C for two days in the dark. After stratification, the pots were transferred into a growth chamber (PGR15; Conviron, Winnipeg, Canada), under 10 h light (150 µE⋅m^−2^⋅s^−1^)/14 h dark at 20°C, 75% relative humidity, and a 584.5‐pascal (Pa) vapor pressure deficit. For the first 10 days, the trays were kept covered with a transparent plastic dome to maintain high humidity for efficient germination and uniform seedling growth. On the 11th day, the seedlings were thinned to make space for a single healthy plant per pot. The plants were bottom‐irrigated alternately with water and 0.5× Hoagland solution twice a week until the start of the experimental stress treatments. For the inoculation, 30‐day‐old plants were used if they had a fully developed rosette and had not transitioned to flowering.

### Preparation of bacterial inoculum

*Pseudomonas syringae* pv. *tomato* DC3000, a bacterium pathogenic to *A. thaliana*, was streaked onto a fresh King’s medium B (KB) (King et al., 1954) (HiMedia) agar (KBA) plate containing rifampicin (50 μg/mL) and grown for 36 h at 28°C. A single bacterial colony was inoculated into 5 mL of KB broth supplemented with rifampicin (50 μg/mL) to initiate a primary culture, which was grown with shaking (200 rpm) at 28°C for 12–15 h. For each batch of 20 plants, 1 L of secondary culture was initiated using 0.5% primary culture (v/v) for use as an inoculum. The bacterial cells were harvested at OD_600_ 0.3–0.4 by centrifugation at 4270 *×* g for 10 min at room temperature, and the bacterial pellet was washed three times with sterile water and resuspended in sterile water. The final concentration of the suspension was adjusted to OD_600_ 0.01. This dilution was further serially diluted and plated onto KBA plates containing rifampicin. The bacteria were counted (colony‐forming units [CFU]), and the OD_600_ at 0.01 was equated to 2.8 × 10^6^ CFU/mL.

### Combined stress imposition

Non‐flowering 30‐day‐old plants were subjected to drought stress. The potting mix around the plants grown in open pots and strip trays was covered carefully from all sides using 3–4 thin rectangular pieces of plastic and clear adhesive tape to prevent the entry of the bacterial suspension into the pot during the inoculation, which could change the water status of the potting mix (Appendix 1). Drought stress was imposed by withholding irrigation. The moisture status of the potting mix (expressed as the field capacity [FC]) was monitored gravimetrically (Ramegowda et al., 2013). The FC at any given fresh weight (FW) was calculated using the formula: FC (%) = [(FW – DW)/(SW − DW)] × 100 (Sinha et al., 2019). By imposing a progressive drought, the plants were brought down from 100% FC (Ψ_w_ = −2.89 megapascal [MPa]) to 40% FC (Ψ_w_ = −3.9 MPa) in seven days. This FC is considered to be a moderately severe stress for *A. thaliana* (Ma et al., 2014; Gupta et al., 2016). On the 37th day of growth, before the inoculations, a 2% agarose solution was poured around the plants in each container type to prevent the entry of the bacterial suspension through small open spaces around the plant and thereby prevent changes to the FC of the potting mix. A 2% agarose solution is easy to pour as it stays in a molten state when it has cooled enough to pour, but then solidifies at room temparature to block spaces well without affecting plant growth and water status.

To inoculate 20 plants, 1 L of bacterial suspension (OD_600_ 0.01, 2.8 × 10^6^ CFU/mL) was poured into an ethanol‐sterilized tray, and the surfactant Silwet L‐77 (Lehle Seeds, Momentive Performance Materials, Waterford, New York, USA) was added to the suspension to a final concentration of 0.01%, just before dipping the plants. The inoculations were performed between 1100 and 1200 hours, 3–4 h after lights were turned on. This was kept the same for all experiments because the time of inoculation and the plant’s circadian clock directly affect the defense responses and stomatal movement (Zhang et al., 2013). The plants were inverted, and their rosettes were dipped into the bacterial suspension for 2 min, gently swirled, and then removed. The plants were allowed to dry for 10 min, then returned to the growth chamber and placed under a plastic dome for 5–6 h to maintain high humidity (80–90%), which is critical as it supports disease development (Xin et al., 2016). The time of inoculation was considered to be 0 days post‐inoculation (dpi). The drought levels were not maintained at 40% FC by adding the required amount of water; the drought was instead allowed to progress, bringing the soil moisture level from 40% FC on 0 dpi to 25% FC by 3 dpi. This allowed the plants to experience combined stress, with the intensity of each stressor increasing with each passing day.

**Figure 4:**
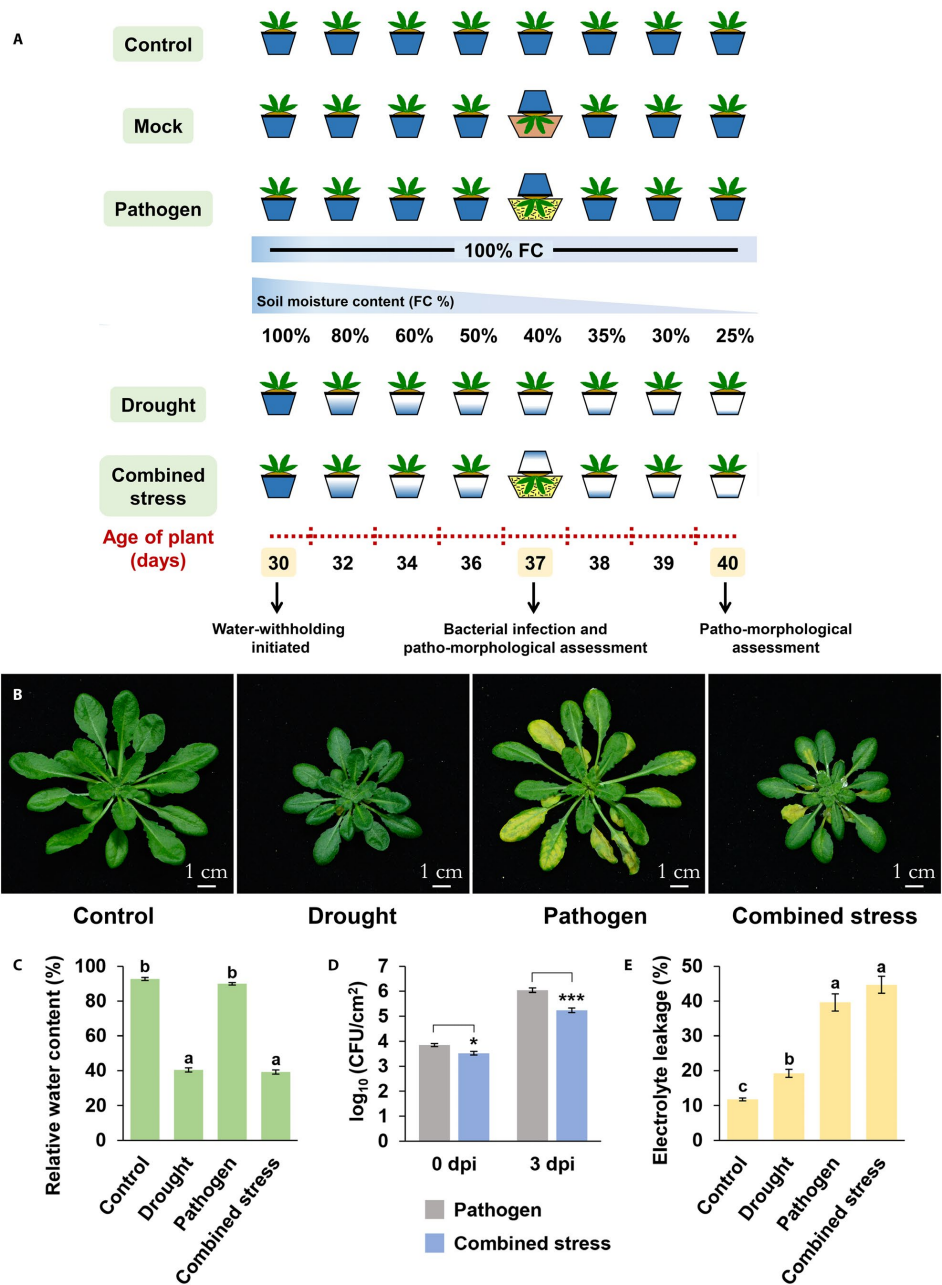
Optimized methodology and physiological observations. (A) Schematic depiction of the methodology optimized for the combined stress treatment. *Arabidopsis thaliana* Col‐0 plants were subjected to a combined drought and bacterial stress. The drought was initiated in 30‐day‐old plants by withholding water. After a further seven days, the potting mix around the plants was covered at 40% field capacity (FC) (Ψ_w_ = −3.9 MPa), and agarose was poured to plug the spaces. The plants were then dipped into a bacterial suspension (2.8 × 10^6^ CFU/mL) for 2 min before being placed back under the original growth conditions to allow the drought to progress further. Patho‐morphological assessments were done at 0 days post‐inoculation (dpi) and 3 dpi. (B) Representative pictures of the plants under control, single‐stress, and combined‐stress treatments at 3 dpi. (C) Leaf relative water contents at 3 dpi (*n* = 10). (D) Bacterial multiplication numbers in plants infiltrated with bacteria at 0 dpi and 3 dpi (*n* = 6). (E) Electrolyte leakage at 3 dpi (*n* = 5). Physiological observations were made in two independent experiments with reproducible results. Error bars show the standard error of the mean. The different letters in C and E indicate significant differences at *P* < 0.05, as determined using a one‐way ANOVA and a Holm–Šidák multiple comparison test. The asterisks in D indicate significant differences, as determined using an unpaired Student’s *t*‐test; *P < 0.05, ***P < 0.001.

We maintained four control groups of plants. Of these, three groups were kept at 100% FC throughout the experiment: (1) a group of uninfected plants (control), (2) a group of uninfected plants that were dipped into sterile water containing 0.01% Silwet L‐77 (mock control), and (3) a group of plants dipped into a bacterial suspension containing 0.01% Silwet L‐77 (pathogen alone). A fourth control group of plants was subjected to progressive drought without the bacterial infection (drought alone). An outline of the individual‐ and combined‐stress treatments is provided in Fig. 4A.

The qualitative and quantitative assessments were performed by photographing the disease development and scoring different physiological parameters at 3 dpi, by which time the drought‐stressed plants had reached 25% FC.

### Evaluation of stress impact on plants

#### Relative water content

To further assess the perception of drought stress by plants at 3 dpi, the leaf relative water content (LRWC) was measured (Choudhary et al., 2017). Two leaf samples were harvested from each plant, with 10 plants used per stress treatment. The FW of each leaf was measured, after which the samples were floated in sterile water for 5–6 h at room temperature before determining the turgid weight (TW). The leaf samples were then oven‐dried for 2–3 days and reweighed to measure the DW. The LRWC was calculated using the formula: LRWC (%) = [(FW − DW)/(TW − DW)] × 100 (Sinha et al., 2019). At 3 dpi, the LRWC reduced from 90% to 40%, and the growth of plants subjected to drought decreased concomitantly (Fig. 4B, C). The LRWC and plant size were comparable between plants subjected to drought alone or the combined stress, indicating that the inoculation did not change the water status of the plant.

#### In planta bacterial multiplication

The in planta bacterial population was quantified 3 h after the inoculation at 0 dpi, as well as at 3 dpi. Two leaf samples were taken from a single plant, and six inoculated plants were used for the estimation. One leaf disk (1.2 cm diameter) was cut out of each leaf sample (using a cork borer; Carewell Instrument, Ambala Cantt, Ambala, Haryana, India), surface‐sterilized in 0.01% H_2_O_2_ for 10 s, and then rinsed in sterile water. Each leaf disk was separately crushed in 1 mL of sterile water using a homogenizer. The homogenate was serially diluted, and 10 μL of each dilution was plated on KBA containing rifampicin (50 μg/mL). The plates were incubated at 28°C for 36 h, after which the bacterial colonies were counted. The in planta bacterial population in CFUs was calculated using the formula: log_10_ (CFU/cm^2^) = (initial volume of homogenate × number of colonies × dilution factor)/(volume plated × area of leaf disk [cm^2^]). The initial load of bacteria was found to be significantly lower in the combined‐stress plants than in well‐watered plants infected with bacteria alone (Fig. 4D). This could be because of the stomatal closure due to drought stress (Melotto et al., 2017). The bacterial load was again determined at 3 dpi, at which time the drought stress had progressed from 40% FC to 25% FC in the combined‐stress plants. The in planta bacterial population was significantly reduced under the combined stress compared with the well‐watered infected plants. Disease‐associated chlorosis, which was prominent in the well‐watered infected plants, was absent in the combined‐stress plants (Fig. 4B). The presence of drought therefore imparts endurance against *P. syringae* infection to *A. thaliana* plants. These results show that, using our optimized methodology, we could successfully co‐impose drought and bacterial stress and demonstrate that severe drought significantly influences disease outcomes by affecting bacterial entry and multiplication.

#### Membrane leakage

Both drought and *P. syringae* infection affect the integrity of the cell membrane, resulting in higher electrolyte leakage, the estimation of which can thus be a valuable parameter for assessing the extent of damage to plants under single‐ and combined‐stress treatments. Electrolyte leakage was quantified for two leaf samples from a single plant, using five plants from each treatment. The quantification was performed as described by Choudhary et al. (2017). One leaf disk (1.2 cm diameter) was punched from each leaf sample and rinsed three times in sterile water for 30 min to remove the electrolytes adhered to the surface and released from the cut ends. The leaf disks were then floated on 5 mL of sterile water with gentle shaking at 60 rpm at room temperature for 8 h, after which the electrical conductivity was measured for each sample using a conductivity meter (LMCM‐20 metal conductivity meter; Labman Scientific Instruments, Chennai, India). The solution along with the samples was autoclaved, allowed to cool, and the conductivity was measured again. Electrolyte leakage was expressed as the percentage ratio of the initial and final readings. The extent of leakage was higher in plants under single‐ and combined‐stress treatments compared with the control plants (Fig. 4E). Although the electrolyte leakage was lower from leaves under drought stress compared with those under the bacterial treatment, it was comparable between the combined‐stress plants and plants infected with bacteria alone. Thus, despite the lower bacterial number, a simultaneous exposure to two stresses was shown to be severely damaging to the plant.

## CONCLUSIONS

To establish an effective methodology for the imposition of combined stresses, we used the well‐established *A. thaliana*–*P. syringae* pathosystem and employed a gradual water deficit and a dip inoculation for bacterial infection, which is the closest laboratory approximation to the normal infection route through the stomata (Whalen et al., 1991; Jacob et al., 2017). The dip‐inoculation method offers several advantages over the other routinely used infection protocols for *P. syringae* inoculation (Katagiri et al., 2002; Yao et al., 2013). It is easy to perform, requires minimal expertise, and utilizes relatively few resources, all of which are commonly available. Unlike flood inoculation (Ishiga et al., 2011; Dixit et al., 2019) and vacuum infiltration (Sinha et al., 2016), it can be used for mature soil‐grown plants and does not require bulky and costly apparatus such as a vacuum chamber, making it a convenient and low‐cost option. To make it amenable for high‐throughput assays, the use of array trays allows the inoculation of 5–50 plants (depending on the size of the array trays) within 2 min, which is less than the time required to syringe‐infiltrate a single plant (approximately 2–3 min). Thus, our method hastens and simplifies the simultaneous inoculation of a large number of plants. The method ensures a uniform bacterial infection and eliminates the chances of mechanical injury to the plant, which is difficult to avoid during syringe infiltration and even spray inoculation.

To closely mimic the field conditions for the drought imposition, water was withheld and the drought was allowed to develop naturally and gradually throughout the experiment. For the combined‐stress experiments, the use of capped containers or the covering of the potting mix around plants grown in open pots meant that the plants could be easily dip inoculated without altering the water status of the potting mix. Covering the soil surface also ensures that the water loss only occurs through transpiration and not by evaporation from the surface. Thus, the experimental set‐up provides ideal conditions for precisely assessing drought‐induced plant responses. This is advantageous when calculating whole‐plant water‐use efficiency and transpiration efficiency in drought studies. Water is an important component that greatly influences disease outcomes during plant–bacteria interactions (Fatima and Senthil‐Kumar, 2017). The syringe infiltration method invariably adds water into the apoplast at the infection establishment stage, which can severely affect the plant–bacteria interaction and skew the conclusions drawn from pathogen and combined‐stress experiments. We successfully showed that the leaf water content of the plants subjected to only drought was comparable to the plants subjected to combined stress, indicating that dip inoculation does not change the leaf water status of drought‐stressed plants. Thus, the methodology is an improvement over the available protocols used for plant–pathogen and combined‐stress studies.

We also showed that the *A. thaliana* combined‐stress plants have significantly lower in planta bacterial growth than their well‐watered infected counterparts. This is consistent with the previously reported observation that a reduced susceptibility to bacterial attack under severe drought stress (40% and 20% FC) was correlated with the enhanced expression of basal defense genes (Gupta et al., 2016). In addition, combined‐stress plants were reported to show increased levels of the defense hormones salicylic acid and jasmonic acid, but a reduction in abscisic acid levels (Gupta et al., 2017). Abscisic acid is known to act antagonistically to the salicylic acid–mediated defense responses and thus, the low levels of abscisic acid were correlated with the enhanced immunity of the plants under severe drought stress.

Importantly, unlike the methods involving syringe infiltration, our protocol can be flexibly used to investigate both the pre‐ and post‐invasive defenses, which are important components impeding the epiphytic growth and entry of bacteria (Melotto et al., 2008). These physical barriers are also considerably affected by various environmental factors such as drought, suboptimal temperature and light, CO_2_, and osmotic stress (Melotto et al., 2017), and are therefore crucial for combined‐stress studies. The method will also be crucial for the study of genotypes in which the plant’s physiology makes it difficult to pressure‐infiltrate the leaves, such as the *suppressor of npr1‐1, constitutive1* (*snc1*) mutant (Zhang et al., 2003) and most of the abscisic acid–deficient mutants (Koornneef et al., 1982), all of which are smaller in size and have curly leaves. This methodology can be further exploited to screen ecotypes and mutant libraries in several plant species, such as tomato (*Solanum lycopersicum* L.), tobacco (*Nicotiana tabacum* L., *Nicotiana benthamiana* L.), and pepper (*Capsicum annuum* L.), to facilitate the development of phenome resources under combined stress and against different foliar pathogens such as species of *Xanthomonas* or *Salmonella*. This technique will therefore benefit plant scientists with wide research interests.

## AUTHOR CONTRIBUTIONS

M.S.‐K. conceived the study. M.S.‐K. and A.C. designed the experiments. A.C. performed the experiments and drafted the manuscript. M.S.‐K. critically reviewed and revised the manuscript. Both authors gave their approval for the publication of the final revised version.

## Figures and Tables

**Figure A1:**
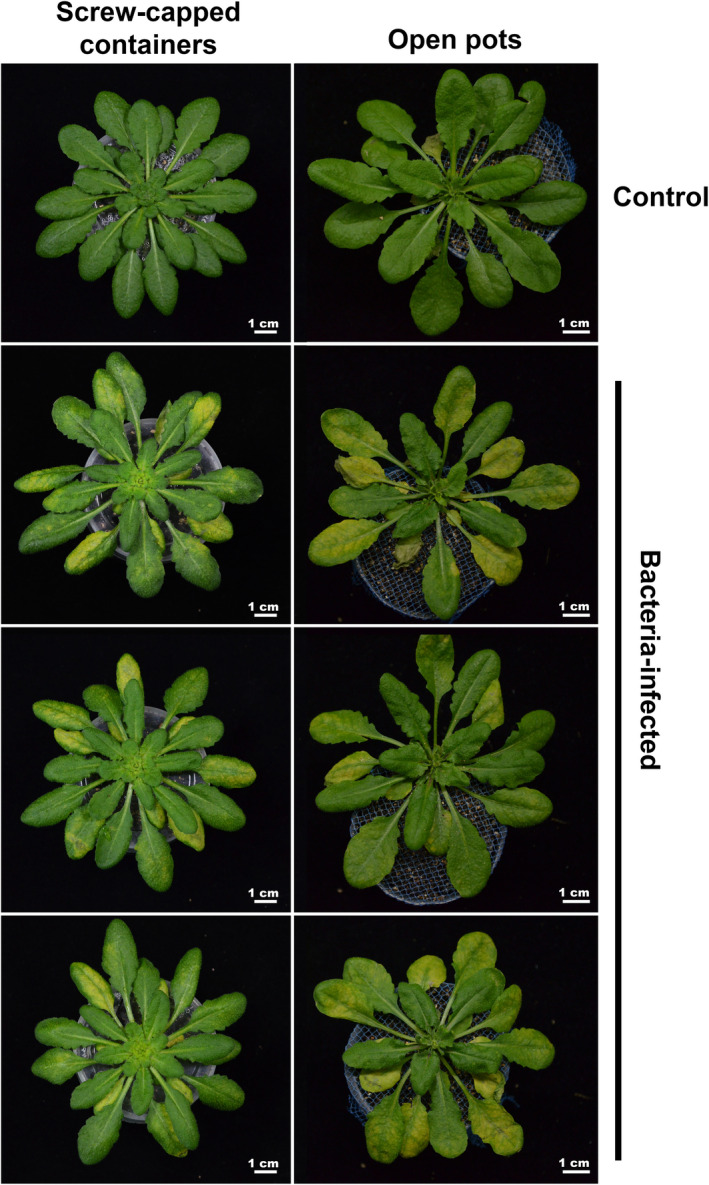
Forty‐day‐old *Arabidopsis thaliana* plants grown in screw‐capped containers (left) and open individual pots (right). Well‐watered 37‐day‐old plants were dipped in a bacterial suspension (2.8 × 10^6^ CFU/mL) for 2 min. Representative images of control plants (one replicate) and bacteria‐infected plants (three replicates) at 3 dpi are shown.

## APPENDIX 1 A detailed summary of the plant growth conditions and the combined stress protocol.

### Materials required

NOTE: Materials and equipment listed here are those used in our laboratory. Equivalent materials can be used as needed.

- Screw‐capped plastic containers (6.0 cm height, 5.2 cm diameter) (Kissan 33; Right Industries, Mumbai, India)
- Individual open plastic pots (4.5 cm height, 5 cm diameter) (Goyal Agri Products, Gurgaon, India)
- Five‐pot strip trays (4.5 cm height, 27 cm length), 24‐pot strip trays (6 cm height, 310 cm length), or 50‐pot strip trays (5 cm height, 53.5 cm length) (Goyal Agri Products)
- Awl
- Portable weighing balance (Kerro Weighing Balance, 0.01 g; Shivam Scientific, Surat, India)
- Agropeat and vermiculite (Varsha Enterprises, Jayanagar, Bengaluru, Karnataka, India)
- Autoclave
- Nylon mesh
- Rubber bands
- Hoagland solution (cat. no. TS1094; HiMedia, Mumbai, India)
- Plastic trays and domes
- Growth chamber (PGR15; Conviron, Winnipeg, Canada)
- King’s medium B base (cat. no. M1544; HiMedia)
- Agarose (cat. no. 50181, SeaKem; Lonza, Basel, Switzerland)
- Plates (90‐mm Petri plates)
- Culture shaker
- Clear adhesive tape
- Black plastic bags (Airsoft Paper & Hygiene Products, Bengaluru, India)
- Silwet L‐77 (Lehle Seeds, Momentive Performance Materials, Waterford, New York, USA)
- 1‐mL micropipette and tips
- Scalpel or scissors

### Methodology

*Plant growth conditions:*

NOTE: Before beginning the experiment, it is important to decide the platform to be used for growing plants. One can use individual pots (with or without a screw cap) or an array tray, which come in several sizes and are suitable for growing 50–100 plants per tray (one plant per pot). Array trays enable the dip inoculation of large numbers of plants in a short time, although it is important to ensure that the rosettes of the plants do not hamper the growth of neighboring plants within the tray. For the drought experiments, all pots should contain equal amounts of potting mix. Individual pots and shorter strip trays (five pots per strip) provide better control over the weighing, which is an important part of the gravimetric measurement of the water status of the potting mix. The use of screw‐capped containers provides an additional advantage as they prevent the growth of fungi and insect pests on the surface of the potting mix, thereby eliminating the need for insecticide or fungicide applications.

1. Before use, use an awl to punch 2–3 holes at the bottom of the small, screw‐capped plastic containers (Fig. 1A) to allow water absorption, and one hole in the cap through which the seeds can be sown. Open plastic pots can be used without modification (Fig. 2A). Cut five‐pot strips from each array tray (Fig. 3A).
2. Use a portable weighing balance and note the weight of each empty container, pot, and strip tray.
3. Prepare the potting mix of 3 : 1 agropeat and vermiculite (v/v). Autoclave and let the potting mix air dry completely.
4. Fill each container, pot, and strip tray with dry potting mix and note their dry weights (DWs). The DW should be the same for all containers/pots/strip trays.
5. Cover the strip trays and open pots with a nylon mesh using rubber bands to secure the potting mix and prevent the potting mix or plant from falling during the dip inoculation.
6. Prepare 0.5× Hoagland solution and pour into a flat tray. Place the containers/pots/strip trays in the flat tray and allow the potting mix to be soaked completely for 2 h. Drain out the excess solution and note the weight of the container/pot/strip tray + saturated potting mix (saturated weight [SW]).
7. Sprinkle *Arabidopsis thaliana* seeds directly into the potting mix.
8. Cover all the trays with a plastic dome and stratify the seeds in the dark at 4°C for two days to ensure synchronized germination.
9. Transfer the seeds in their trays into a growth chamber, with 10 h light (150 µE⋅m^−2^⋅s^−1^)/14 h dark at a temperature of 20°C, a relative humidity of 75%, and a 584.5‐Pa vapor pressure deficit (calculated using http://cronklab.wikidot.com/calculation‐of‐vapour‐pressure‐deficit [accessed 20 October 2020]).
10. Keep the plants covered with a transparent plastic dome for 10 days after transferring them to the growth room to maintain high humidity, which promotes efficient germination and uniform seedling growth.
11. Remove the plastic dome on the 11th day. Thin out excess plants to leave one healthy plant per pot.
12. Bottom‐irrigate the plants alternately with water and 0.5× Hoagland solution twice a week until the start of the experiment. Do not overwater the plants or allow the potting mix to dry out between irrigations.

NOTE: Plants should not transition to the flowering stage until the end of the experiment. Any that do so should be discarded.

*Preparation of the bacterial suspension:*

NOTE: The following procedure is for the inoculation of 20 plants. The volume of bacterial culture should be scaled depending on the number of plants being used.

1. Four days before the inoculations, take a glycerol stock of the virulent strain of the pathogen *Pseudomonas syringae* pv. *tomato* DC3000 and streak it on a fresh King’s medium B (KB) agar (KBA) plate containing rifampicin (50 μg/mL). Incubate for two days at 28°C.
2. Use a single bacterial colony to initiate a 5‐mL primary culture in KB broth supplemented with rifampicin (50 μg/mL). Place the culture vial on a shaker at 200 rpm at 28°C for 12–15 h.
3. Using the primary culture, initiate a 1‐L secondary culture in KB broth supplemented with rifampicin (50 μg/mL). It is advisable to produce sufficient secondary culture to ensure sufficient inoculum for infection despite the loss of bacterial cells during the subsequent washing steps. Place the culture vial on a shaker at 200 rpm at 28°C for 5–6 h.
4. When the OD_600_ of the secondary culture reaches 0.3–0.4, harvest the bacterial cells by centrifugation at 4270 *×* g for 10 min at room temperature. Wash the bacterial pellet three times in sterile water.
5. Resuspend the final pellet in sterile water. Measure the OD_600_ and adjust the final concentration of the suspension to 0.01 using sterile water. It is advisable to prepare the final bacterial suspension in a large beaker for use in all the inoculations. This prevents variations in the concentrations of different suspensions between different inoculations.
6. Serially dilute 1 mL of the final suspension in sterile water and plate 10^−^^4^ dilution on KBA plates (with 50 μg/mL rifampicin). Incubate for two days at 28°C and count the number of bacterial colonies to calculate the colony‐forming units (CFUs) in the suspension at OD_600_. An OD_600_ of 0.01 was equated to 2.8 × 10^6^ CFU/mL in the described protocol.

*Combined stress imposition:*

1. Before initiating the drought, cover the potting mix around the 30‐day‐old plants grown in strip trays and open pots from all sides using a thin black plastic bag and clear adhesive tape. This is important to prevent the entry of the suspension into the pot, which could change the water status of the potting mix. Take caution while covering to avoid inflicting physical injury to the plant. Capped containers offer an advantage here as this step can be avoided.
2. Impose drought stress on 30‐day‐old plants by withholding irrigation.

NOTE: This methodology can be flexibly used for 2–5‐week‐old *A. thaliana* plants. The time required for the moisture content of the potting mix or soil to decrease to a particular level depends on several factors (described below). It is important to standardize the age of the plants at the time of drought initiation because this can strongly influence the response of the plant to both drought and bacterial infection (Fig. A1). The plants must not transition to the flowering stage.

1. Gravimetrically monitor the decreasing moisture level of the potting mix (expressed as field capacity [FC]) by weighing the pots every day and calculating the FC at any given fresh weight (FW) using the formula: FC(%)=[(FW‐DW)/(SW‐DW)]×100.

NOTE: To minimize the variation in the FC between pots/trays, the factors that can affect water depletion should be closely monitored. Filling the pots with equal amounts of completely dried potting mix rather than wet potting mix minimizes the fluctuations in the weight of each pot after water saturation. Covering the potting mix with plastic or using screw‐capped containers prevents an uneven evaporative loss during the experiment. In addition, using plants with similar growth and leaf areas helps to keep transpiration loss uniform across replicates. The pot weight, as well as the water potential at 100% FC at the time of water withholding and at 40% FC (or desired soil moisture status) at the time of inoculation, should be noted across the replicates to ensure uniformity.

1. It takes seven days for the plants used here to decrease from 100% FC (Ψ_w_ = −2.89 MPa) to 40% FC (Ψ_w_ = −3.9 MPa) (on the 37th day).

NOTE: This timeline is specific and will vary with the size of the container, open or closed state of the container, composition of potting mix, plant genotype, age of the plant, and number of plants in each pot. The duration must therefore be strictly standardized before undertaking the experiment. In addition, soil moisture status is not a universal measure of drought severity. An FC of 40% (Ψ_w_ = −3.9 MPa) is a moderately severe stress for *A. thaliana*, but it may not be the same for a drought‐tolerant species; therefore, care must be taken before concluding the drought severity at a particular FC for each plant species.

1. On the 37th day, before the inoculations, pour a small blob of 2% agarose around the plants grown in the containers/pots/strip trays using a 1‐mL micropipette tip cut using a scalpel or scissors. This must be done on the day of inoculation and not before because the agarose plug loses moisture over time. Therefore, we observed that if the plug is poured earlier than the day of inoculation, it shrivels and fails to cover the area around the plant. This step is important because, despite using plastic to cover the potting mix, even a small open space around the plant can be sufficient for the suspension to enter and change the FC of the potting mix. Agarose works better here because it does not solidify immediately once it cools, allowing researchers to pour it around plants without causing heat shock.
2. Pour 1 L of inoculum from the prepared homogenous bacterial suspension (OD_600_ 0.01, 2.8 × 10^6^ CFU/mL) into an ethanol‐sterilized plastic tray for dipping 20 *A. thaliana* plants. Add the surfactant Silwet L‐77 to the suspension to a final concentration of 0.01%, just before dipping the plants.

NOTE: The size of the container can be varied to suit the platform used for growing the plants. Trays work better for strip trays, while a wide‐mouth beaker can be used for single pots.

NOTE: The concentration of the bacterial inoculum should be standardized according to the experiment. A lower concentration of bacteria is generally recommended for transcriptomic studies. A very high concentration can even cause exaggerated disease occurrence, which may lead to a sudden collapse of the plants and diminished differences between the genotypes or the conditions being compared.

1. Invert the plants and immerse only the rosettes completely into the suspension for 2 min. Swirl the plants gently to avoid inflicting physical damage to the plants. It is easy to hold two strip trays (one in each hand) or four single pots/containers (two in each hand) at the same time; thus, it takes only 2 min for a person to inoculate 4–10 plants together. This compares favorably with syringe infiltration, which requires 3–4 min (or more for droughted plants, owing to their closed stomata) to pressure‐infiltrate a single plant.

NOTE: Plants should be divided into batches of equal number, and each batch should be dipped in an equal amount of inoculum taken from the same initially prepared bacterial suspension to ensure uniformity.

1. Following inoculation, allow the plants to dry for 10 min, then return them to the growth chamber under their original growth conditions.

NOTE: The bacterial culture should be packed well and autoclaved before disposal.

1. Keep the plants under a transparent plastic dome for 5–6 h to maintain high humidity, which is critical for disease development. The day of inoculation is considered to be 0 days post‐inoculation (dpi). Determine the initial in planta pathogen load (see Methods section) in plants subjected to the combined stress or bacterial stress alone.

NOTE: The initial in planta pathogen load should be measured immediately within 1–2 h of bacterial inoculation. Surface sterilization of the leaves is essential in the case of dip inoculation, as the bacteria residing on the leaf surface can skew the calculations for the in planta bacterial number.

1. Remove the domes after 6 h, and continue withholding water for the drought‐stressed plants. Allow the drought to progress naturally until the end of the experiment. Weigh the containers before and after the inoculation to monitor the moisture content of the potting mix until the end of the experiment. The moisture status of the potting mix can be measured gravimetrically by weighing the pots and by taking water potential measurements, and the drought being experienced by the plant at any given time can be calculated by measuring the leaf water potential.

NOTE: The FC on the day of inoculation and the nature of drought to be imposed (to be maintained or allowed to progress post‐inoculation) can vary with the research question associated with the experiment.

1. Maintain four groups of control plants: (1) a group of uninfected plants kept at 100% FC throughout the experiment (control), (2) a group of uninfected plants kept at 100% FC throughout the experiment and dipped in sterile water containing 0.01% Silwet L‐77 (mock control), (3) a group of plants kept at 100% FC throughout the experiment and dipped in a bacterial suspension containing 0.01% Silwet L‐77 (pathogen alone), and (4) a group of uninfected plants subjected to drought stress alone (drought alone).
2. At 3 dpi, determine the final in planta pathogen load in plants subjected to the combined stress and bacterial stress alone. Estimate the extent of membrane leakage and the leaf water content in plants under single‐ and combined‐stress treatments. Record disease development by taking pictures of the whole plants.

NOTE: This methodology also has the potential to be used for root phenotyping studies. *Arabidopsis thaliana* roots, being very soft, are a bit difficult to work with, but the root architectures of tomato, tobacco, and chickpea can be studied using this methodology. For these species, it is easy to dislodge the soil or potting mix from the plastic containers without damaging the main root and lateral roots. Depending upon the experiment and the plant being used, the size of the container can be varied. This will be useful not only for drought studies but also for studying the plant’s response against rhizospheric pathogens.

NOTE: The methodology allows the effective co‐imposition of drought and bacterial stress, and can also be used in labs studying wider aspects of plant–bacterial interactions and plant‐defense responses using routine bacterial infection assays.
